# A New Staging System Based on the Dynamic Prognostic Nomogram for Elderly Patients With Primary Gastrointestinal Diffuse Large B-Cell Lymphoma

**DOI:** 10.3389/fmed.2022.860993

**Published:** 2022-05-02

**Authors:** Junmin Wang, Weirui Ren, Chuang Zhang, Xiaoya Wang

**Affiliations:** ^1^Department of Gastroenterology, The Third Hospital of Hebei Medical University, Shijiazhuang, China; ^2^Department of Pediatric Surgery, The Second Hospital of Hebei Medical University, Shijiazhuang, China; ^3^Jitang College of North China University of Science and Technology, Tangshan, China

**Keywords:** primary gastric diffuse large B-cell lymphoma, prognostic nomogram, overall survival, dynamically predict, elderly patients

## Abstract

**Objective:**

The purpose of this study is to establish an accurate prognostic model based on important clinical parameters to predict the overall survival (OS) of elderly patients with primary gastrointestinal diffuse large B-cell lymphoma (EGI DLBCL).

**Methods:**

The Cox regression analysis is based on data from the Surveillance, Epidemiology, and End Results (SEER) database.

**Results:**

A total of 1,783 EGI DLBCL cases were eligible for the study [median (interquartile range, IQR) age, 75 (68–82) years; 974 (54.63%) males], of which 1,248 were randomly assigned to the development cohort, while 535 were into the validation cohort. A more accurate and convenient dynamic prognostic nomogram based on age, stage, radiation, and chemotherapy was developed and validated, of which the predictive performance was superior to that of the Ann Arbor staging system [C-index:0.69 (95% CI:0.67–0.71) vs. 56 (95%CI:0.54–0.58); *P* < 0.001]. The 3- and 5-year AUC values of ROC curves for 3-year OS and 5-year OS in the development cohort and the validation cohort were were alll above 0.7.

**Conclusion:**

We establish and validate a more accurate and convenient dynamic prognostic nomogram for patients with EGI DLBCL, which can provide evidence for individual treatment and follow-up.

## Introduction

Primary gastrointestinal (GI) lymphoma is the most common type of extranodal lymphoma, accounting for approximately 25% of all primary extranodal lymphomas (1). However, primary gastrointestinal lymphoma accounts for only 1–4% of all gastrointestinal cancers (2). More than half of the cases occur in the stomach, followed by the small intestine and ileocecum (2). Histopathological findings showed the following types: marginal zone lymphoma (MALT), diffuse large B-cell lymphoma (DLBCL), enteropathy-associated lymphoma (EATL), mantle cell lymphoma (MCL), etc. According to histological type, DLBCL is the most common gastrointestinal lymphoma, with an estimated prevalence of 40–50% (2, 3).

Since 1993, the International Prognostic Index (IPI) has long been used for risk stratification, which can provide prognosis prediction and treatment guidance for patients with diffuse large B-cell lymphoma (DLBCL). The five factors of IPI score include Ann Arbor Stage III/IV, age > 60 years, elevated lactate dehydrogenase (LDH), Eastern Cooperative Oncology Group (ECOG) working status (PS) of ≥ 2, and at least one extranodal location are involved. The sum allows patients to be divided into four independent groups through each point of four factors, with a 5-year overall survival rate (OS) of 26–73% (4). GI DLBCL is usually diagnosed as low or medium IPI. Among the five factors of IPI score, age plays a non-negligible role in patients with GI DLBCL (5). Also, the National Comprehensive Cancer Network (NCCN) guidelines include the Ann Arbor staging of primary diffuse large B-cell lymphoma to guide clinical treatment and follow-up (6). As the conventional staging system for non-Hodgkin’s lymphoma (NHL), the Ann Arbor staging system considers the location of lymph node spreading as the basis for staging (7). It does not include other factors that may affect long-term survival, such as age and depth of tumor invasion. Also, the Ann Arbor staging system is not considered the best staging system for primary gastric diffuse large B-cell lymphoma (8). Elderly patients (≥60 years old), with primary gastrointestinal diffuse large B-cell lymphoma (EGI DLBCL), have a significantly higher risk of death compared with the adult population because they are much more likely to receive palliative rather than curative care. At the same time, elderly patients have poor physical condition and many complications and are less resistant to comprehensive treatment (9, 10). Therefore, it has a clinically significant role in predicting the survival of EGI DLBCL.

Therefore, based on the latest population-based data from Surveillance, Epidemiology, and End Results (SEER) database, this study investigated a large number of patients to develop a survival prediction nomogram and a web-based survival rate calculator that can dynamically predict the long-term survival of EGI DLBCL.

## Materials and Methods

### Data Collection

This study is a retrospective cohort study using the largest publicly available data set on the human cancers’ SEER database. The database contains information collected from different cancer registries in 18 geographic regions of the United States, which currently account for approximately 27.8% of the total population of the United States (11). The SEER database has a wide population coverage and high data accuracy. Since any information in the SEER database does not require the explicit consent of the patient, our research is not subject to the ethical permission requirements of the institutional review board, and informed consent was obtained from all participants.

### Patient Selection

The inclusion criteria for this study are as follows: (1) Patients with pathological diagnosis of primary gastric diffuse large B-cell lymphoma from 2010 to 2015. (2) Pathological results support the diagnosis of the patient; (3) patients with ≥ 60 years old. Patients who meet any of the following criteria are excluded: (1) Ann Arbor staging was unknown; (2) Missing demographic information, including race, and marital status; (3) Missing clinical and treatment information; and (4) Missing survival information. In total, 1,783 elderly patients (≥ 60 years old), with primary gastric diffuse large B-cell lymphoma (EGI DLBCL), were included in the final analysis, randomly assigned to the development and validation cohorts with a ratio of 7:3.

### Statistical Analysis

Overall survival (OS), measured from the date of diagnosis to the time of death from any cause or last follow-up, was employed as the outcome of interest. The Wilcoxon-Mann-Whitney test or Fisher’s exact test was performed to measure the distribution differences of variables between the development and validation cohorts, where appropriate. Hazard ratios (HRs) and 95% confidence intervals (CIs) were calculated by the univariate Cox regression model to quantify the effect of potential prognostic predictors on OS. Then, predictors significantly associated with OS in the univariate analysis were entered into the multivariate analysis to validate their significance by applying a backward procedure based on the Akaike information criterion (AIC) (12).

A dynamic prognostic nomogram for OS was then generated based on the identified independent prognostic factors. Harrell’s concordance index (C-index) was used to assess the discrimination performance and compared between the dynamic prognostic nomogram and the Ann Arbor staging system. Calibration curves were adopted as indicators of internal calibration by plotting the nomogram-predicted probabilities against the observed probabilities *via* a bootstrap method with 1,000 resamples (13, 14). In the validation cohort, nomogram performance was assessed using the same methods as in the development cohort.

We further utilized the quartile to determine the optimal cut-off value for the total risk score (derived from the nomogram) and established a prognostic risk stratification to allocate patients of EGI DLBCL into groups at different risks. By comparing the prognostic score and the survival curve of the staging system grouping with the ROC curve at a specific time point, the discriminating power of the model was evaluated. In addition, the comparison of prediction effectiveness was measured using the receiver operating characteristics (ROC) curve at a specific time point between nomogram and a single meaningful variable (15). The above analyses were all performed using R software (version 3.6.3), and the statistically significant difference was determined to be two-sided *P* < 0.05.

## Results

A total of 1,783 EGI DLBCL cases were eligible for the study [median (interquartile range, IQR) age, 75 (68–82) years; 974 (54.63%) males], of which 1,248 were randomly assigned to the development cohort, while 535 were into the validation cohort. The demographical and clinical characteristics of patients in the development and validation cohorts were all similar (Table 1). The median follow-up in the development cohort was 27 months (IQR, 6–77 months); the OS for 3- and 5-year were 50.68 and 45.44%, respectively. The median follow-up time in the validation cohort was 28 months (IQR, 5–78 months); 3- and 5-year OS rates were 50.56 and 42.05%, respectively.

**TABLE 1:** Patient characteristics and clinicopathological variables.

Characteristic	No. of patients (%)		*P*-value
	
	Development cohort (*n* = 1,248)	Validation cohort (*n* = 535)	
Age (years)			0.731
≤75	627 (50.24)	265 (49.53)	
76–82	341 (27.32)	141 (26.36)	
≥83	280 (22.44)	129 (24.11)	
Sex			0.223
Male	670 (53.69)	304 (56.82)	
Female	578 (46.31)	231 (43.18)	
Year of diagnosis			0.626
2004–2006	370 (29.65)	175 (32.71)	
2007-2009	322 (25.80)	130 (24.30)	
2010–2012	304 (24.36)	128 (23.93)	
2013–2015	252 (20.19)	102 (19.07)	
Race			0.658
White	1,027 (82.29)	432 (80.75)	
Black	65 (5.21)	33 (6.17)	
Others	156 (12.50)	70 (13.08)	
Marital status			0.313
Married	723 (57.93)	321 (60.00)	
Divorced/separated	93 (7.45)	49 (9.16)	
Widowed	301 (24.12)	119 (22.24)	
Single	131 (10.50)	46 (8.60)	
Ann Arbor stage			0.470
I	613 (49.12)	244 (45.61)	
II	248 (19.87)	112 (20.93)	
III	98 (7.85)	51 (9.53)	
IV	289 (23.16)	128 (23.93)	
Chemotherapy			0.779
No	304 (24.36)	127 (23.74)	
Yes	944 (75.64)	408 (76.26)	
Radiotherapy			0.826
No	1,016 (81.41)	438 (81.87)	
Yes	232 (18.59)	97 (18.13)	
Surgery			0.150
No	1,127 (90.30)	471 (88.04)	
Yes	121 (9.70)	64 (11.96)	
Tumor location			0.062
Upper third	138 (11.06)	79(14.77)	
Mid and low third	374 (29.97)	134 (25.05)	
Overlapping stomach	130 (10.42)	50 (9.35)	
Stomach (NOS)	606 (48.56)	272 (50.84)	

**FIGURE 1:**
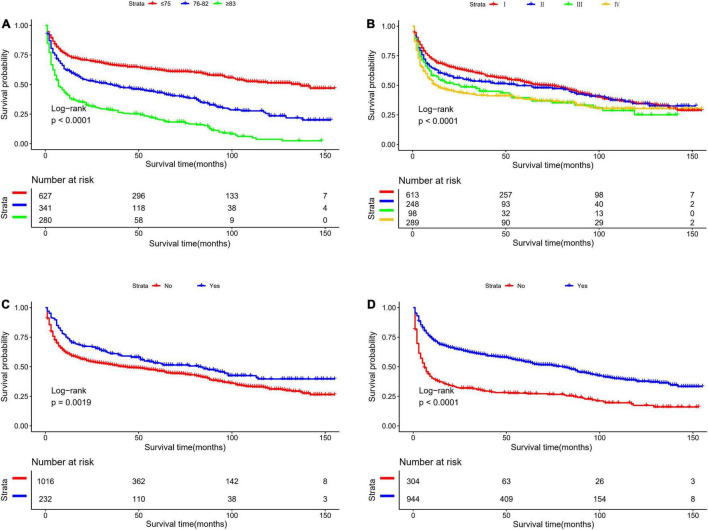
Kaplan-Meier survival curves for overall survival in the development cohort, as stratified by **(A)** Age, **(B)** stage, **(C)** radiation, **(D)** chemotherapy.

Table 2 presents the results of univariate and multivariate analyses to include the nomogram variables for OS in the development cohort. In the univariate Cox model, except for sex, race, surgery, and tumor location, the remaining variables were significantly associated with OS. In the multivariate Cox model, all selected variables by applying the AIC-based backward procedure (age, stage, radiation, and chemotherapy) had significant effects on OS (all *P* < 0.05). At the same time, the survival curves were drawn separately for the meaningful variables of multivariate regression (Figure 1).

**TABLE 2:** Results of the univariate and multivariate cox models, including the nomogram variables for overall survival in the development cohort.

Characteristic	Univariate analysis		Multivariate analysis	
		
	HR(95%CI)	*P*-value	HR(95%CI)	*P*-value
Age (years)				
≤ 75	Reference		Reference	
76–82	1.89 (1.58–2.27)	<0.001	1.97 (1.64–2.36)	<0.001
≥ 83	3.49 (2.92–4.17)	<0.001	3.27 (2.72–3.93)	<0.001
Sex				
Male	Reference			
Female	0.95 (0.82–1.10)	0.470		
Year of diagnosis				
2004–2006	Reference			
2007–2009	0.89 (0.74–1.07)	0.201		
2010–2012	0.77 (0.63–0.95)	0.013		
2013–2015	0.79 (0.62–1.00)	0.045		
Race				
White	Reference			
Black	1.14 (0.84–1.57)	0.398		
Others	0.87 (0.69–1.10)	0.247		
Marital status				
Married	Reference			
Divorced/separated	0.95 (0.70–1.30)	0.764		
Widowed	1.72 (1.46–2.03)	<0.001		
Single	0.93 (0.72–1.21)	0.606		
Ann Arbor stage				
I	Reference		Reference	
II	1.11 (0.91–1.35)	0.318	1.33 (1.09–1.63)	0.005
III	1.38 (1.06–1.81)	0.018	1.69 (1.29–2.22)	<0.001
IV	1.48 (1.24–1.78)	<0.001	1.79 (1.48–2.16)	<0.001
Chemotherapy				
No	Reference		Reference	
Yes	0.44 (0.38–0.52)	<0.001	0.49 (0.41–0.58)	<0.001
Radiotherapy				
No	Reference		Reference	
Yes	0.74 (0.61–0.90)	0.002	0.77 (0.63–0.94)	0.009
Surgery				
No	Reference			
Yes	0.96 (0.75–1.23)	0.736		
Tumor location				
Upper third	Reference			
Mid and low third	1.23 (0.94–1.62)	0.128		
Overlapping stomach	1.23 (0.89–1.71)	0.209		
Stomach (NOS)	1.17 (0.90–1.51)	0.236		

*HR, hazard ratio; CI, confidence interval.*

**FIGURE 2:**
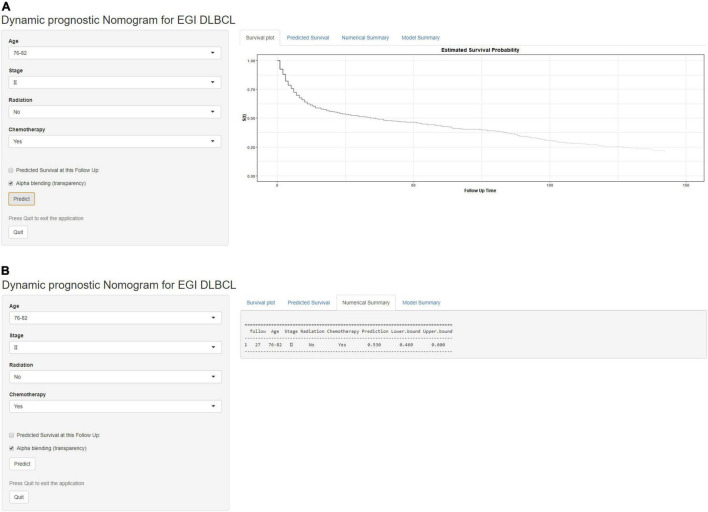
A web-based dynamic prognostic nomogram for overall survival (OS) estimation in elderly patients with primary gastrointestinal diffuse large B-cell lymphoma (EGI DLBCL). **(A)** Patient with an Ann Arbor stage of III, age at 76–82 years, no radiation, who received chemotherapy according to the web survival rate calculator (95% CI 46–60%). **(B)** 95% confidence interval (CI) according to the web survival rate calculator.

**FIGURE 3:**
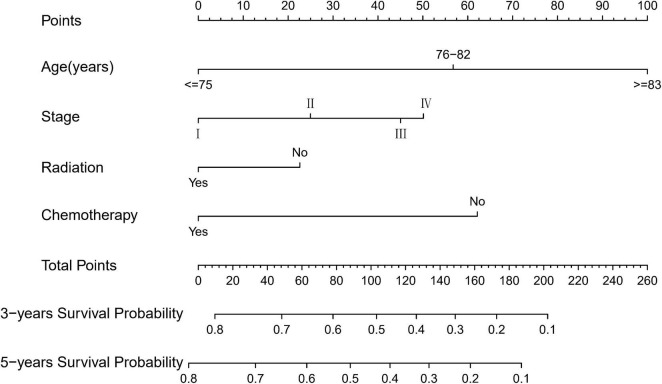
Graphical nomogram scoring system for overall survival.

**FIGURE 4:**
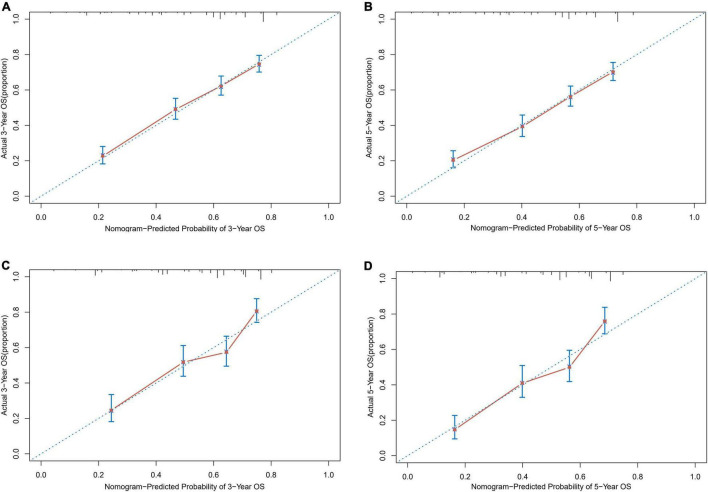
Calibration plots of survival probabilities in patients with EGI DLBCL. **(A)** At 3-year and **(B)** 5-year in the development cohort. **(C)** At 3-year and **(D)** 5-year in the validation cohort.

Based on the proven independent prognostic factors, a user-friendly and accurate dynamic prognostic nomogram^1^ was established (Figure 2A); a point assignment for each factor and total risk score calculation for each patient is described in detail in Supplementary Table 1 and Figure 3. The dynamic prognostic nomogram would conveniently provide the accurately predicted probability of OS based on the total risk score, which was calculated automatically according to the input characteristics of subjects (Figure 2B). The C-indices of the nomogram in the development cohort and the validation cohort were 0.69 (95% CI, 0.67–0.71) and 0.69 (95% CI, 0.66–0.72), respectively. Figure 4 shows the nomogram calibration curves for 3- and 5- years in the development cohort and validation cohort. Calibration plots revealed that the nomogram was well-calibrated, with the superb agreement between the nomogram-predicted probabilities and the observed probabilities of 3- and 5-year OS.

Table 3 shows the comparison of the dynamic prognostic nomogram and the Ann Arbor staging system concerning prognostic accuracy for OS in patients of EGI DLBCL. The C-indices of the nomogram were significantly higher than those of the Ann Arbor staging system ([-index (95% CI) for the development cohort, 0.56 (0.54–0.58); C-index (95% CI) for the validation cohort, 0.54 (0.51–0.58)], with the *P*-values both less than 0.001 for the development cohort and the validation cohort.

**TABLE 3:** C-indices of the nomograms and Ann Arbor staging system in elderly patients with primary gastrointestinal diffuse large B-cell lymphoma (EGI DLBCL).

Items	Development cohort			Validation cohort		
		
	No. of patients	C-index (95%CI)	*P*-value*^a^*	No. of patients	C-index (95%CI)	*P*-value*^a^*
All patients with EGI DLBCL	1,248			535		
Ann Arbor staging system	–	0.56 (0.54–0.58)	Reference	–	0.54 (0.51–0.58)	Reference
Dynamic Nomogram	–	0.69 (0.67–0.71)	<0.001	–	0.69 (0.66–0.72)	<0.001

*^a^P-value indicates the difference in the C-indices.*

**FIGURE 5:**
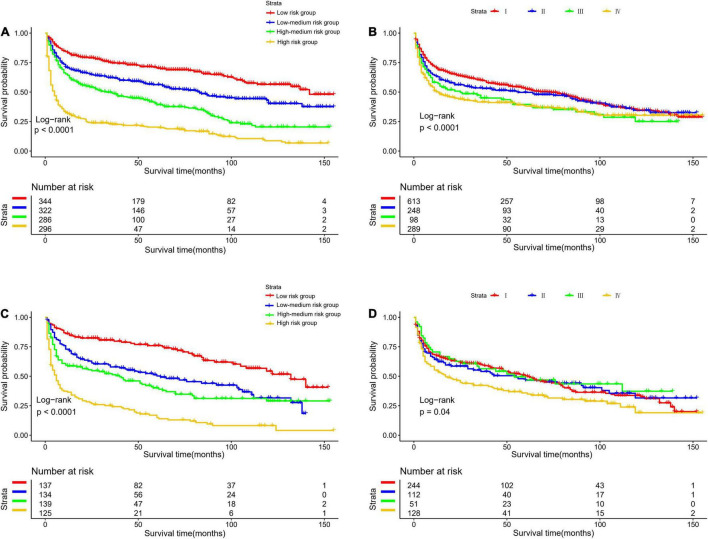
Kaplan-Meier survival curves for risk stratification in the development cohort **(A)** nomogram **(B)** stage and validation cohort **(C)** nomogram **(D)** stage.

**FIGURE 6:**
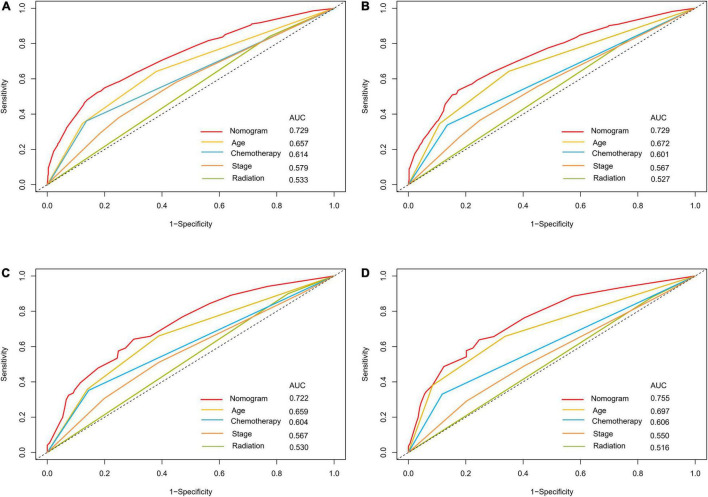
ROC curves for predictions of overall survival in the development cohort **(A,C)** and validation cohort **(B,D)** at 3, 5-year.

The optimal cut-off value for the total risk score was calculated based on the quartile of the patient’s prognostic score. By comparing the survival curves between the quartiles of risk scores and the Ann Arbor staging, we can see that the difference between the survival curves of the quartiles in the development cohort and the validation cohort is significantly better than that of the Ann Arbor staging (Figure 5). In addition, we compared the 3- and 5-year ROC curves of the nomogram in the development cohort and the validation cohort with a single multivariate meaningful variable at specific time points. The results show that the predictive power of the nomogram is higher than that of a single multivariate meaningful variable (Figure 6).

## Discussion

Primary gastric lymphoma (PGL) is a rare tumor that accounts for 2–8% of all gastric tumors (16). DLBCL accounts for 45–50% of all primary gastric lymphomas (PGL). To the best of our knowledge, the present population-based study is the first time to establish and validate a dynamic prognostic nomogram for survival prediction in patients of EGI DLBCL, which can incorporate sociodemographic and clinicopathologic characteristics. Importantly, the dynamic prognostic nomogram was further utilized to generate individualized risk stratifications to further individualize the choice of the treatment strategy.

Precision medicine has developed rapidly in recent years. Clinicians must formulate personalized treatment and follow-up strategies for patients, which requires more accurate and convenient survival models. The line graph integrates tumor staging and multiple prognostic factors into a simple and practical tool, which has been widely used to predict the long-term survival of patients with malignant tumors. The accuracy is usually better than traditional tumor staging systems (17–21). In addition, to increase the convenience of the prediction model, some scholars have used a web-based calculator to predict the long-term survival of cancer patients (21–23). Primary gastric diffuse large B-cell lymphoma is a rare malignant tumor, and its incidence is less than 5% of gastric malignant tumors (24). There is no previous study on a web-based calculator that can be used to predict the survival rate of elderly patients with primary gastric diffuse large B-cell lymphoma. Therefore, this study included 1,783 patients in the SEER database to analyze factors affecting long-term survival and used a survival rate calculator based on the nomogram to dynamically predict the prognosis of elder patients with primary gastric diffuse large B cell lymphoma, which can determine individual treatment and follow-up strategies.

To the best of our knowledge, this is the largest retrospective case series of major EGI DLBCL, which aims to obtain a prognostic model for predicting OS (25–29). In this study, we developed a nomogram to predict the prognosis of patients with EGI DLBCL based on the following four important factors: age at diagnosis, Ann Arbor staging, chemotherapy, and radiotherapy. In the present study, based on an AIC-backward procedure, we identified four independent predictors for OS, which were in keeping with the previous findings and could be easily obtained from routine clinical measurements. By incorporating these four independent predictors, we successfully established and validated a dynamic prognostic nomogram for OS, with calibration curves showing good accordance between nomogram-based and actual overall survival probability. In this study, the 5-year survival rate of patients 76–82 years old and in stage II, who received chemotherapy without radiotherapy, was 53% (95% CI, 46.0–60.0%). Compared with other web-based survival rate calculators, web-based calculators can provide better visualization. It can dynamically predict the cancer-specific survival rate of patients with EGI DLBCL at different time points and help identify patients at high risk of cancer-specific death. Because of its easy-to-use clinical applicability, we do believe this tool would be widespread acceptance and serves as a practical calculator in routine clinical practice. The clinicians could calculate the variable scores and sum them according to the values of 4 meaningful variables for each patient, and then predict the possible incidence of different survival periods based on the total score.

In the past few decades, several staging systems have been developed to improve the prognostic stratification of NHL. The Ann Arbor staging system is widely used in NHL staging. The Lugano staging system is an improved version of the original Ann Arbor staging system designed for the staging of GI lymphoma. The purpose of this staging system is to combine the depth of invasion and the measurement of distal lymph node invasion. The most widely used classification is the Lugano staging system, which has been adopted by the eighth edition of the Cancer Staging Manual of the American Joint Committee on Cancer (30). The SEER database only provides data on the Ann Arbor staging. Our survival analysis shows that Ann Arbor staging is an independent prognostic indicator of GI DLBCL. When compared with the Ann Arbor staging system, the dynamic prognostic nomogram showed superior prognostic accuracy, with both higher C-indices for the nomogram in EGI DLBCL.

Despite these strengths, this study still has several limitations that need to be taken into consideration. First and most importantly, it is regrettable that the SEER database could not provide information about the extranodal extension (ENE) in patients with EGI DLBCL, thus, we fail to reassess them according to the eighth edition of the Ann Arbor staging system. Further study is encouraged to overcome this shortcoming. Second, the SEER database lacks information on overall comorbidity and lifestyle habits, which may impact the prognosis of patients and lead to a change in subsequent therapeutic decisions. Accordingly, the dynamic prognostic nomogram, to some extent, may be limited by the failure to include these prognostic predictors. Finally, although the precise and easy-to-use dynamic prognostic nomogram was established with the latest data from a large population-based US database, we did not perform external validation, leaving its clinical applicability unknown in non-US population, and our prediction model needs an independent validation in different population before any clinical application.

## Conclusion

We establish and validate a more accurate and convenient dynamic prognostic nomogram for patients with EGI DLBCL, which can provide evidence for individual treatment and follow-up. Further independent studies are warranted to externally evaluate and validate our dynamic prognostic nomogram.

## Data Availability Statement

The datasets presented in this study can be found in online repositories. The names of the repository/repositories and accession number(s) can be found in the article/Supplementary Material.

## Ethics Statement

Ethical review and approval was not required for the study on human participants in accordance with the local legislation and institutional requirements. Written informed consent for participation was not required for this study in accordance with the national legislation and the institutional requirements. Written informed consent was not obtained from the individual(s) for the publication of any potentially identifiable images or data included in this article.

## Author Contributions

WR and JW conceived and designed the study. All authors wrote the article, downloaded, screened the data from the SEER database, participated in analyzing the data, read, and approved the final manuscript.

## Conflict of Interest

The authors declare that the research was conducted in the absence of any commercial or financial relationships that could be construed as a potential conflict of interest.

## Publisher’s Note

All claims expressed in this article are solely those of the authors and do not necessarily represent those of their affiliated organizations, or those of the publisher, the editors and the reviewers. Any product that may be evaluated in this article, or claim that may be made by its manufacturer, is not guaranteed or endorsed by the publisher.

## Footnotes

1
https://junminwang.shinyapps.io/DynNomapp-EGIDLBCL/
