# Supplementary material for: A New Staging System Based on the Dynamic Prognostic Nomogram for Elderly Patients With Primary Gastrointestinal Diffuse Large B-Cell Lymphoma
Source: Front Med (Lausanne). 2022 May 2;9:860993. doi: 10.3389/fmed.2022.860993 (PMC9108771; doi:10.3389/fmed.2022.860993)
Supplement: Supplementary file 1 [file Table_1.doc]

**Supplementary Table 1** Overall survival nomogram scoring system^a^

| **Age(years)** | **Points** | **Stage** | **Points** | **Radiation** | **Points** |
| --- | --- | --- | --- | --- | --- |
| ≤75 | 0 | Ⅰ | 0 | Yes | 0 |
| 76-82 | 56.75 | Ⅱ | 24.96 | No | 22.61 |
| ≥83 | 100.00 | Ⅲ | 45.04 |  |  |
| 50 | 16 | Ⅳ | 50.13 |  |  |
| 60 | 23 |  |  |  |  |
| 70 | 37 |  |  |  |  |
| 80 | 58 |  |  |  |  |
| 90 | 79 |  |  |  |  |
| 100 | 100 |  |  |  |  |
|  |  |  |  |  |  |
| **Chemotherapy** | **Points** | **Total Points** | **3-year OS** | **Total Points** | **5-year OS** |
| Yes | 0 | 203.8 | 0.1 | 185.3 | 0.1 |
| No | 62.13 | 172.1 | 0.2 | 157.6 | 0.2 |
|  | 9 | 148.6 | 0.3 | 133.5 | 0.3 |
|  | 13 | 127.2 | 0.4 | 111.8 | 0.4 |
|  |  | 103.6 | 0.5 | 88.2 | 0.5 |
|  |  | 78.5 | 0.6 | 62.7 | 0.6 |
|  |  | 48.2 | 0.7 | 32.1 | 0.7 |
|  |  | 8.9 | 0.8 |  |  |

^a^ OS predictions corresponding to total points not shown in the table may be obtained by linear interpolation.
